# Survivorship and complications of cementless compared to cemented posterior-stabilized total knee arthroplasties: A systematic review and meta-analysis

**DOI:** 10.1051/sicotj/2024017

**Published:** 2024-05-30

**Authors:** Esfandiar Chahidi, Sagi Martinov, Filip Simion, Camille Mercier, Liam Sabot, Theofylaktos Kyriakydis, Antoine Callewier, Jacques Hernigou

**Affiliations:** 1 Université Libre de Bruxelles Av. Franklin Roosevelt 50 1050 Brussels Belgium; 2 Orthopaedic Department, EpiCURA Baudour, Hornu, Ath Hospitals Rue Louis Caty 136 7331 Saint-Ghislain Hainaut Belgium; 3 2nd Department of Orthopaedic Surgery and Traumatology, Aristotle University of Thessaloniki, “G. Gennimatas” General Hospital Thessaloniki Hellas Greece; 4 Laboratoire de Biochimie Osseuse et Métabolique ULB, Bone and Metabolic Biochemistry Research Laboratory, Université Libre de Bruxelles Lenniksebaan 808 1070 Brussels Belgium

**Keywords:** Cementless total knee arthroplasty, Posterior stabilized, Survivorship

## Abstract

*Purpose*: Controversy exists on the best fixation for total knee arthroplasty (TKA). Non-cemented fixation has been theorized to improve patient outcomes and longevity of implantation but no study has focused on comparison between cemented or cementless posterior-stabilized implants despite being the most commonly or second most frequently utilized implant in most total knee replacement registries. *Methods*: Inclusion criteria with observational and interventional papers, and review articles that focused on patients with cementless and cemented PS TKAs were used to analyze outcomes such as implant survivorship, complication, or revision rates. Using a combination of keywords, a systematic search was performed on Medline (PubMed), Embase, and Cochrane Library for Meta-Analysis. *Results*: When using the specified criteria, only 8 studies were selected for full-text analysis and meta-analysis after eliminating screening duplicates, titles, and abstracts without full-text access. These eight studies contain 1652 patients, 693 in the non-cemented Group, and 959 in the cemented total knee prosthesis Group. The meta-analysis revealed the advantage of cementless fixation over cemented fixation in implant survivorship, with 0.6% and 2.6% of aseptic loosening in each Group. The cumulative survival at 12 years was 97.4% for the cementless Group and 89.2% for the cemented Group. The subgroup with a stem showed a positive outcome for cementless fixation over cemented fixation regarding implant survivorship. No differences between the cemented and cementless TKAs were observed in patient-reported outcomes, revision rates, or radiolucent line development. *Conclusion*: We observed comparable rates for cemented and cementless posterior-stabilized TKAs over a medium-term follow-up period.

## Introduction

The advent of total knee arthroplasty (TKA) has revolutionized the management of end-stage knee osteoarthritis, offering patients a chance at an improved quality of life and mobility [1]. Cemented TKAs have long been considered the gold standard, with 80%–90% of all TKAs performed [2, 3]. In the past decade, the reasons for revising total knee prostheses have evolved. Previously, polyethylene wear was the main cause of revision, but now aseptic loosening is the leading reason with 24% of indications of revision of TKA compared to 2% for polyethylene wear [2, 4–6]. Hence, finding alternative anchoring techniques is becoming increasingly crucial. As a result, modern cementless prostheses, with porous metal (close to the porosity of bone) at the bone/prosthesis interface provide a more suitable biological environment for the bone tissue growth interface. Biological fixation through bone ingrowth and ongrowth, continuous remodeling at the bone-implant interface preventing fatigue failures, avoidance of cement-related complications, easier revision, and improved operating room efficiency make cementless fixation an attractive option [7]. These prostheses have shown encouraging results concerning their lifespan in various recent publications, with ten-year follow-up data providing valuable insights [8–10].

However, these studies mainly report the results of unconstrained prostheses. Among the different types of TKA, posterior-stabilized (PS) constraints TKA is popular due to its potential for increased stability [11]. Currently, it is one of the most widely implanted TKA, representing 20%–50% of implants in implant registries [2, 3]. The posterior-stabilized designs are classified as partially constrained compared to cruciate retaining implants, as they aim to substitute the PCL with a post-cam system to provide a more stable component interface [11]. PS TKA has demonstrated excellent lifetime survivorship in cemented TKA. The main issue when using these prostheses lies in the contact occurring at the post-cam interface, which causes polyethylene wear at the level of the cam mechanism and results in higher stress on the polyethylene insert itself [11]. Therefore, the use of a PS design in cementless TKA remains controversial due to unpredictable results from stresses placed on the tibial interface from the cam/post interaction. This interaction was thought to potentially cause excessive micromotion that may inhibit biological fixation and result in early aseptic loosening [12].

To address the knowledge gap of the survival of the most commonly used implant (PS design) in cementless prosthetic knee surgery, we conducted a systematic review and meta-analysis of existing studies on the survivorship and complications of cementless compared to cemented PS TKAs.

## Material and method

Meta-analysis was recorded on the PROSPERO database on 31/01/2023 with registration number: 395962.

The selection criteria focused on studies comparing cementless and cemented PS TKAs and reporting on outcomes such as implant survivorship, complication, or revision rates. Including registry reports like the Belgian Knee Arthroplasty Registry and the National Joint Registry added robustness to the data.

### Bibliographic research protocol

The Cochrane Library, PubMed, EMBASE, and Ovid databases were searched electronically for comparative studies on posterior-stabilized cemented and cementless total knee arthroplasty between January 2000 and December 2022.

### Eligibility articles and search strategy with information sources

Inclusion articles included observational and interventional papers, and review articles published in English that focused on patients with posterior-stabilized cemented and cementless total knee arthroplasty. We also examined the references of the included studies to ensure optimized research.

The following key words were used: (“total knee arthroplasty” [All Fields], OR “TKA” [All Fields], OR “total knee replacement” [All Fields], OR “TKR” [All Fields], OR “knee arthroplasty” [All Fields], OR “knee replacement” [All Fields], OR “cemented” [All Fields], or “cement” [All Fields], OR “uncemented” [All Fields], OR “uncement” [All Fields], OR “cementless” [All Fields], OR “posterior-stabilized” [All Fields], OR “postero-stabilized” [All Fields], OR “postero-stabilised” [All Fields], OR “posterior-stabilised” [All Fields].

### Inclusion criteria

Studies were included according to the PICOS criteria.

*Participants*: Patients with osteoarthritis (OA) or rheumatoid arthritis (RA) of the knee who underwent TKA for the first time, regardless of age, sex and weight.

*Interventions*: Patients received either cementless (both tibial and femoral components) or cemented (both components) total knee arthroplasties, with posterior-stabilized design. All approaches and prosthesis brands were included.

*Outcomes*: Long-term survival rate of prosthesis (with any reason for revision as endpoint), Incidence of radiolucent line, and/or medical complications rates (detailed).

*Study design*: All comparative studies in English.

Using standardized forms, duplicates were removed, and then titles and abstracts were screened by two reviewers (EC and JH). They then thoroughly evaluated the full texts of the potential studies to confirm their eligibility.

### Data extraction and synthesis of results

The two authors (EC and JH) gathered descriptive raw data from selected studies, including first author, publication year, study design, sample size, follow-up time, and outcome measures. Any disagreements were resolved through discussion with a third author. The outcome measures included radiolucent lines, aseptic loosening, total complications, and reoperations. If data was missing or could not be extracted study was excluded. A short narrative synthesis of results was undertaken, and the included articles were divided according to the inclusion criteria

### Bias risk assessment

The risk of bias in the included studies was evaluated independently by authors EC and JH using Cochrane tools for both randomized (RoB-2) and non-randomized studies (ROBINS I). In case of disagreement, SM was consulted to reach a consensus. The potential risk of bias was rated as “low”, “high”, or “unclear” for each trial.

### Statistics

Data analysis was conducted using SPSS 29 software (SPSS Inc., Chicago, USA). The risk difference (RD) and its 95% confidence interval (CI) were used as the effect analysis statistic for binary variables. A fixed model was applied to analyze the risk difference, as all studies were comparative cementless versus cemented total knee arthroplasties with posterior-stabilized components. Heterogeneity was analyzed using a Chi^2^ test (*α* = 0.1) and the *I*^2^ statistic. Heterogeneity was classified as low (<50%), moderate to high (50%–75%), or high (>75%) based on the *I*^2^ value. Subgroup analysis was performed for sensitivity analysis to address any clinical heterogeneity. A *p*-value < 0.05 was considered to indicate a significant difference.

## Results

### Selection of sources of evidence and flow chart

**Figure 1:**
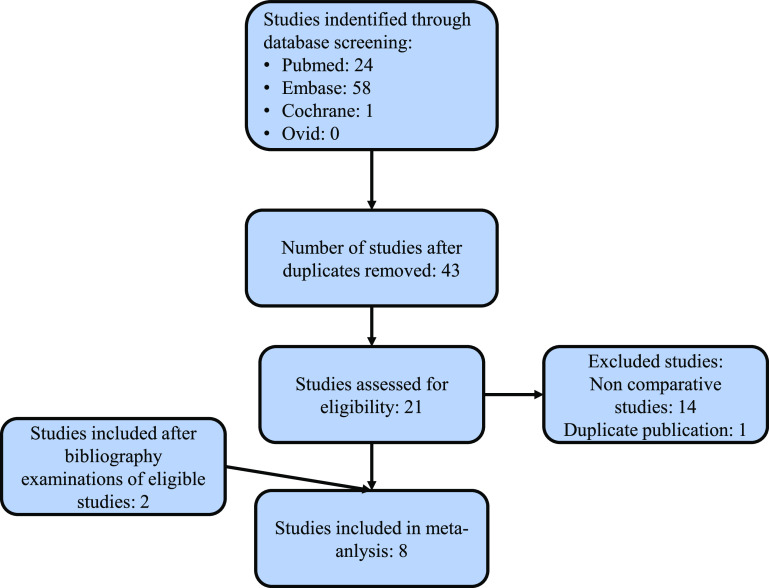
Flowchart illustrating the 2020 PRISMA (Preferred Reporting Items for Systematic Reviews and Meta-Analyses) guidelines for selecting studies in this meta-analysis.

The literature search resulted in 83 articles when using the specified criteria. Only six studies were selected for full-text analysis and meta-analysis after eliminating screening duplicates, titles, and abstracts without full-text access. The bibliography analysis added two more studies, making a total of 8 citations that met the inclusion criteria for the analysis [1, 13–19]. Two studies were RCT [13, 14] and six were non-RCT [1, 15–19]. A summary of this process is provided in Figure 1, depicting the flow diagram of the selection process of studies eligible for the meta-analysis.

### Study characteristics

Table 1 presents the characteristics of the included studies. These eight studies contain 1652 patients, 693 in the non-cemented Group, and 959 in the cemented total knee prosthesis Group. All patients were evaluated between 2003 and 2021, mainly in the USA, Canada, France, Australia, and Japan. Implants used were Triathlon, NexGen, Attune, and New Wave TKAs. One study did not mention the name of the implant used [13].

**Table 1:** Characteristics of the included studies.

Study	Design	Country	Time of inclusion	*N* Cless	*N* Cem	Obesity	Stem	Follow-up	Implants
Sinicrope 2019	Retrospective	USA	2004–2010	108	85	45	1	6	Triathlon
Prudhon 2017	Retrospective	France	2003–2006	100	100	–	1	12	New Wave
Sheridan 2022	Retrospective	Canada	2018–2019	45	147	31,6	1	2.5	Triathlon
Nivbrant 2020	Prospective – RCT	Australia	–	45	47	–	–	2	–
Pulido 2015	Prospective – RCT	USA	2003–2006	106	126	32	0	5	NexGen
Kamath 2011	Retrospective	USA	–	100	312	–	0	5	NexGen
Bagsby 2016	Retrospective	USA	2008–2013	144	97	45	1	3,5	Triathlon
Mikashima 2022	Retrospective	Japan	2020–2021	45	45	25	1	1	Attune

### Assessment of the methodological quality

**Figure 2:**
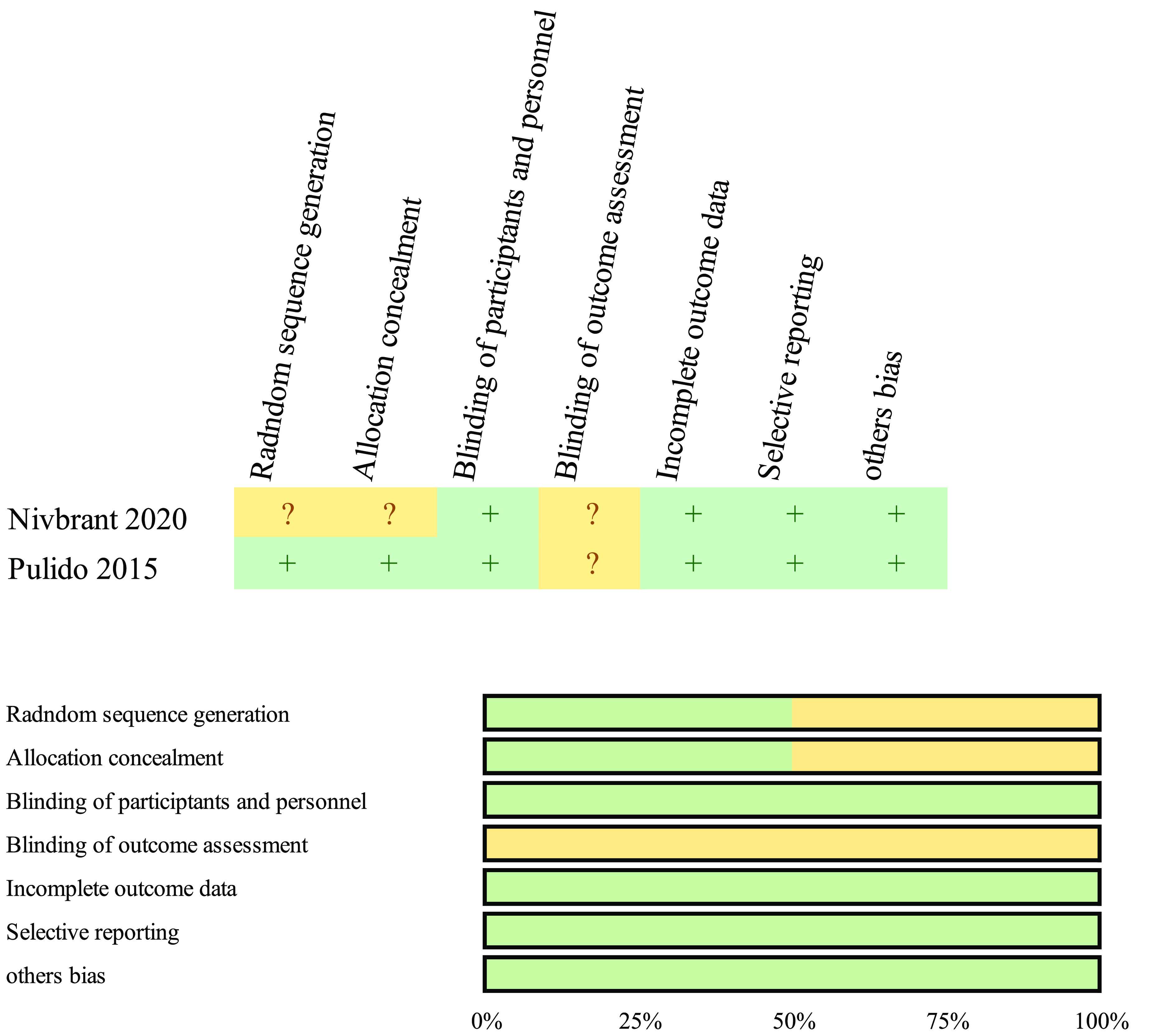
Risk of bias chart and overview displaying the authors’ assessments for each bias risk factor, expressed as percentages utilizing the Rob-2 Cochrane bias evaluation instrument for randomized controlled trials (RCTs).

**Figure 3:**
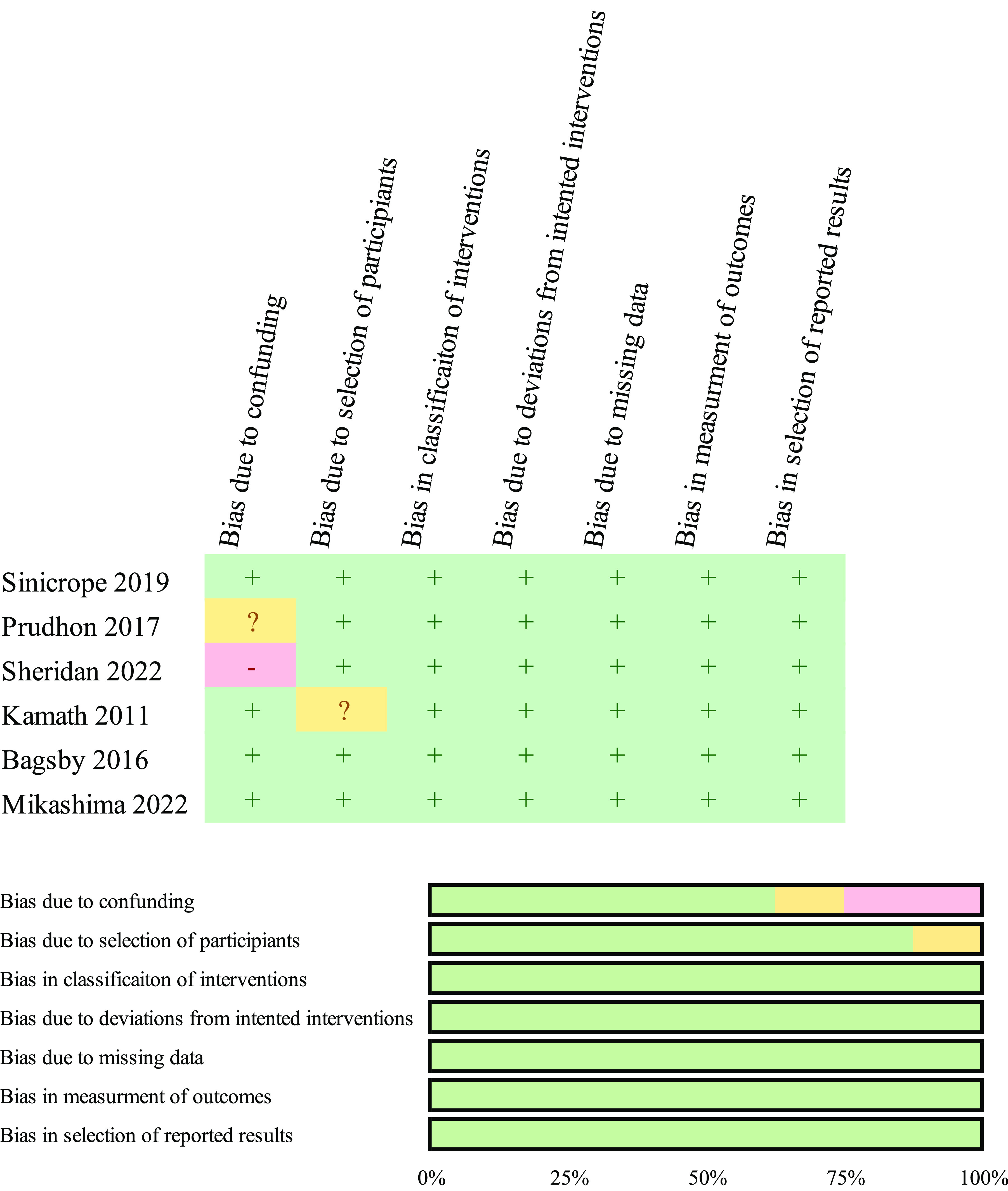
Risk of bias chart and overview displaying the authors’ assessments for each bias risk factor, expressed as percentages utilizing the ROBINS-I Cochrane bias evaluation instrument for non-randomized controlled trials.

Figures 2 and 3 present the responses to individual questions within each domain for the eight studies examined through the Cochrane RoB-2 and ROBINS-I tools. All eight studies exhibited a low likelihood of bias in most domains and a moderate risk of bias in some items, resulting in a low risk for each study.

### Meta-analysis of aseptic loosening

**Figure 4:**
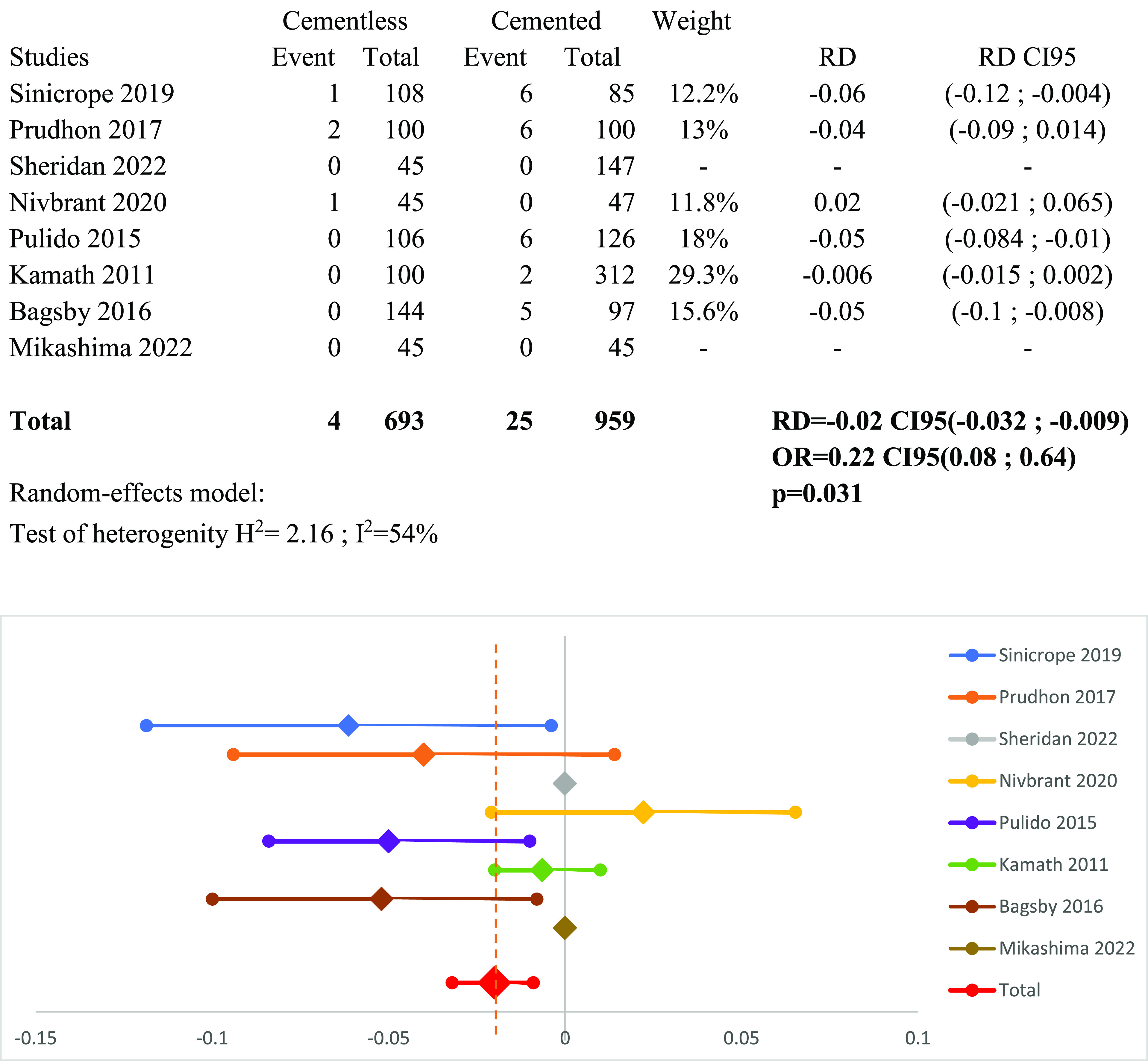
Forest plot demonstrating the risk difference of aseptic loosening between the cementless and cemented groups. RD = Risk Difference; CI = Confidence Interval; OR = Odd Ratio

All the included studies reported rates and delays of aseptic loosening. Heterogeneity was significant, so a random effect model was applied. The meta-analysis revealed the advantage of cementless fixation over cemented fixation in implant survivorship, with 0.6% and 2.6% of aseptic loosening in each Group. The risk difference of RD_AL_ = −0.02 (95% CI: −0.032 to −0.009, *p* = 0.031) is represented in Figure 4.

**Figure 5:**
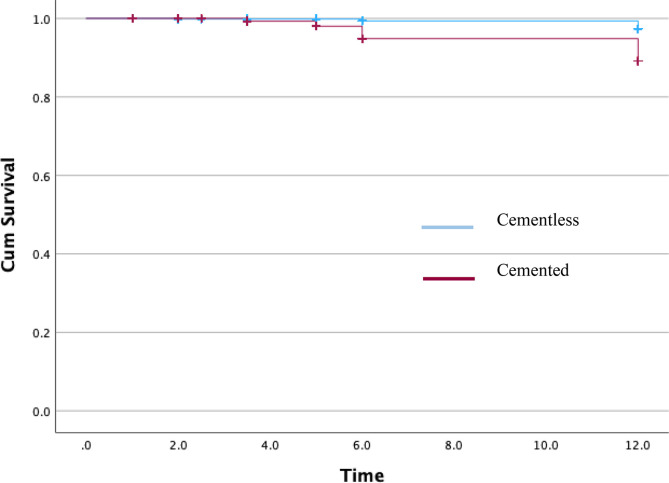
Kaplan–Meyer curve of aseptic loosening between cementless and cemented TKA (*p* = 0.01 – log-rank).

A Kaplan–Meier analysis of cemented and cementless implants using available describes the distribution of loosening over time. We merged all the 1652 patients into a single database for analysis, including the type of implant (cemented vs. cementless), survivorship status (aseptic loosening or not), and time of loosening based on the mean follow-up reported in each study. The cumulative survival at 12 years was 97.4% for the cementless Group and 89.2% for the cemented Group (*p* < 0.01) (Figure 5).

### Subgroup stem versus no stem (Figure 6)

A subgroup analysis was conducted based on the tibial component design in TKA (with or without a stem). One study was excluded from this subgroup analysis as it did not mention the type of tibial implant they used [13]. A random-effect model was used (*I*^2^ = 55%). The subgroup with a stem showed a positive outcome for cementless fixation over cemented fixation regarding implant survivorship, with RD_stem_ = −0.029 (95% CI: −0.05 to −0.01, *p* < 0.001). However, no significant difference was found in the subgroup without a stem, with RD_no-stem_ = −0.02 (95% CI: −0.03 to −0.006, *p* = 0.33).

**Figure 6:**
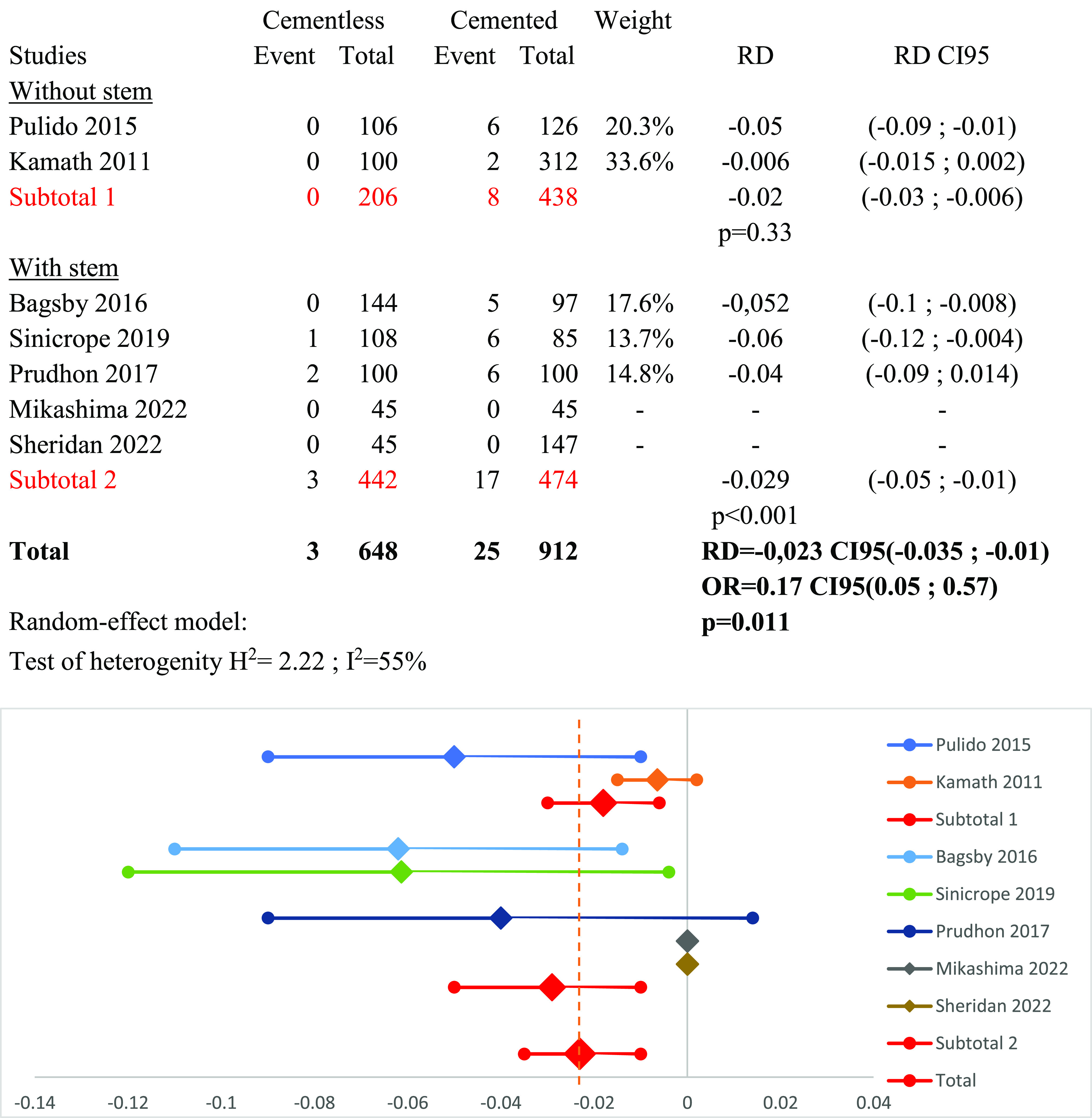
Forest plot demonstrating the risk difference of aseptic loosening between the cementless and cemented groups categorized by the design of the implant: with or without stem. RD = Risk Difference; CI = Confidence Interval; OR = Odd Ratio.

### Subgroup obesity versus general population (Figure 7)

A subgroup analysis was conducted based on the population studied, either obese or non-obese, using a random-effect model (*I*^2^ = 54%). Two studies explored outcomes of cementless PS TKA in overweight patients [17, 19]. The mean BMI of patients in these two studies was 45 kg/m^2^. The mean BMI for the four other studies was 33 kg/m^2^. Two studies did not mention the BMI of their patients [13, 16] and were excluded from this subgroup analysis. The subgroup analysis of obese individuals showed a positive outcome for cementless fixation over cemented fixation regarding implant survivorship, with RD_obesity_ = −0.057 (95% CI: −0.092 to −0.02, *p* = 0.002). No significant difference was found in the general population subgroup, with RD_no-obesity_ = −0.01 (95% CI: −0.02 to 0.001, *p* = 0.25).

**Figure 7:**
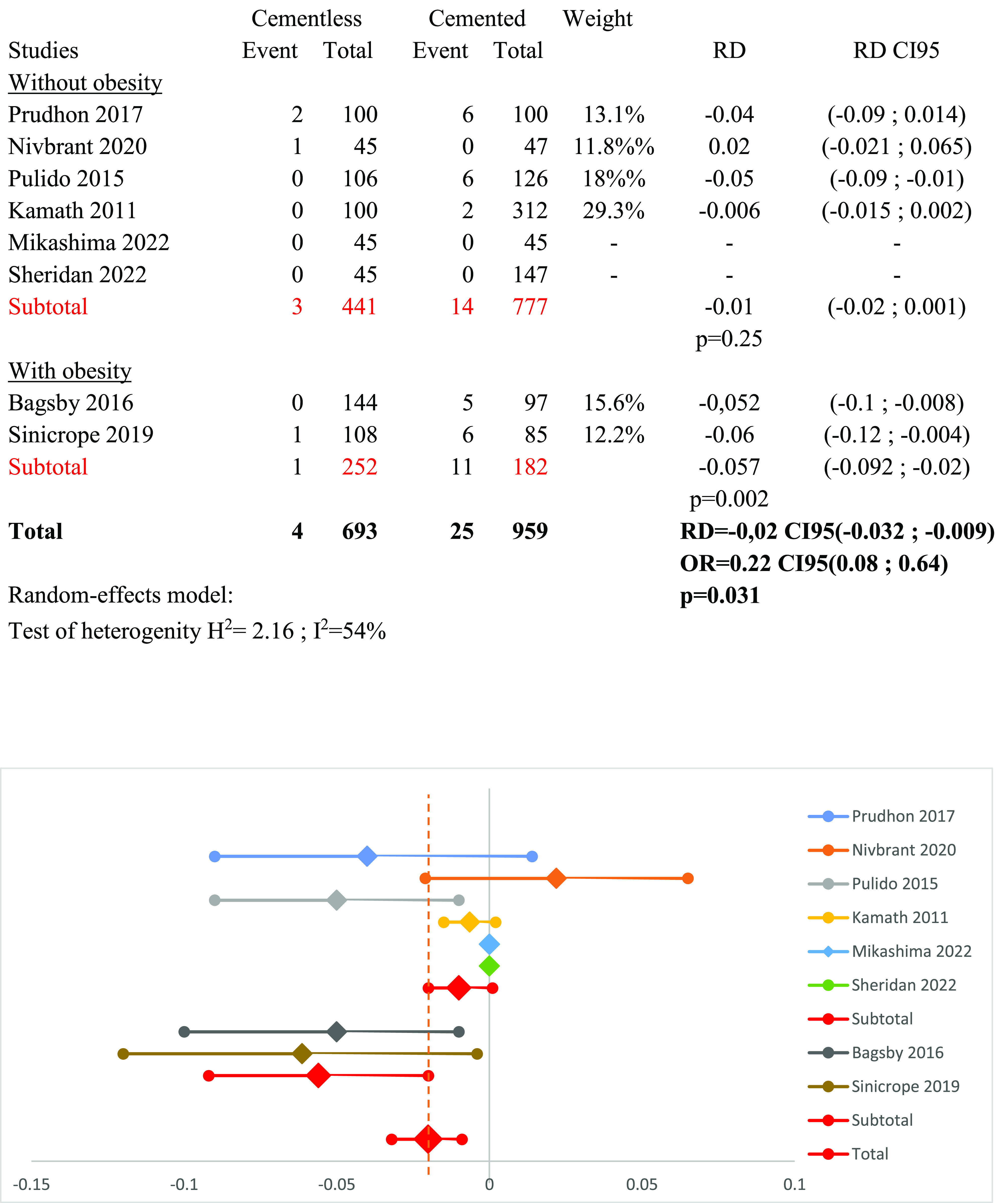
Forest plot demonstrating the risk difference of aseptic loosening between the cementless and cemented groups according to the presence of obesity. RD = Risk Difference; CI = Confidence Interval; OR = Odd Ratio.

### Subgroup RCT versus non-RCT studies (Figure 8)

A subgroup analysis was carried out based on the design of the study (randomized controlled trials (RCTs) versus non-RCT studies), using a fixed effect model. The results showed a risk difference RD_RCT_ = −0.03 (95% CI: −0.06 to 0.002, *p* = 0.65) for RCT studies and RD_no-RCT_ = −0.02 (95% CI: −0.03 to −0.006, *p* = 0.05) for non-RCT studies.

**Figure 8:**
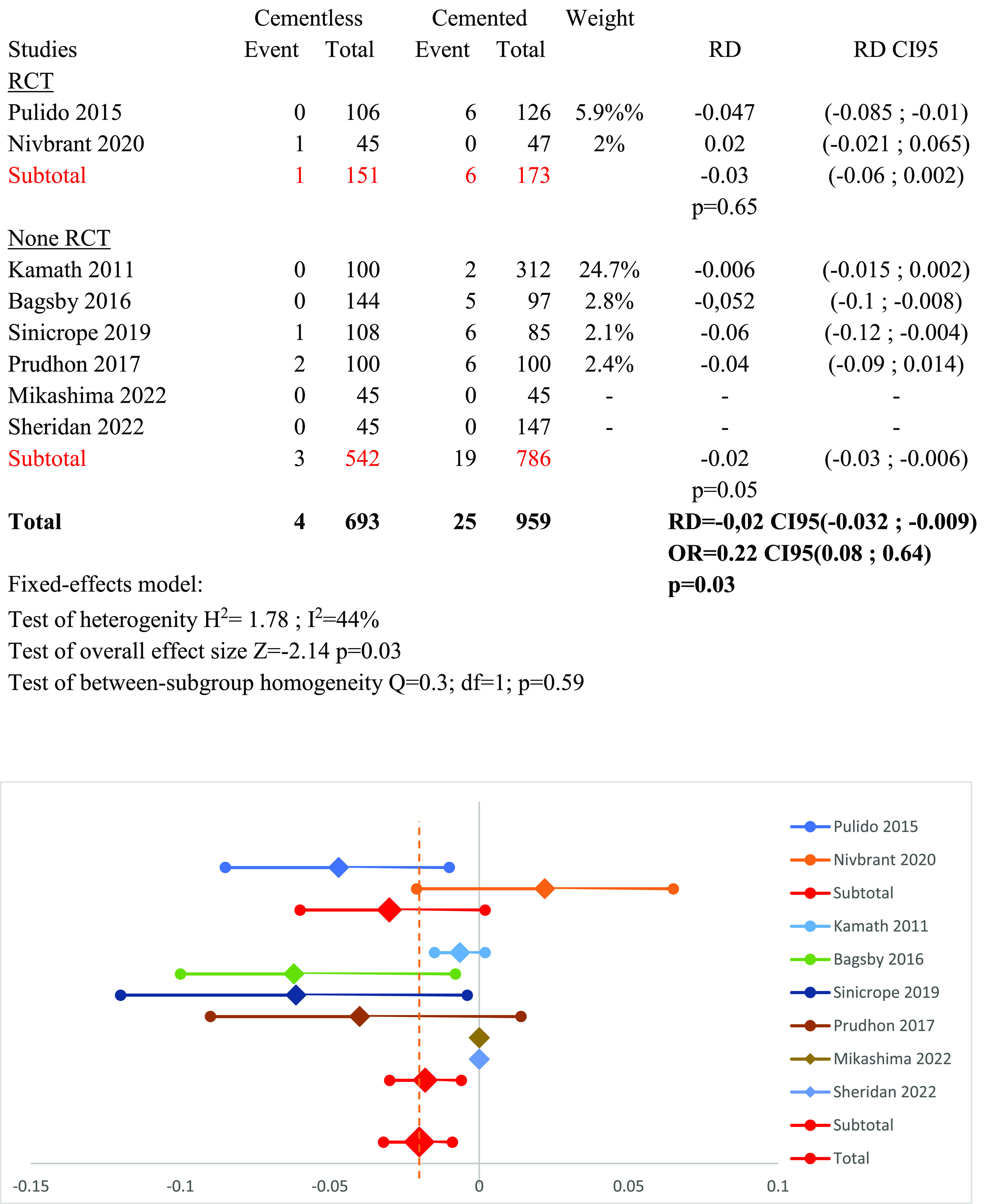
Forest plot demonstrating the risk difference of aseptic loosening between the cementless and cemented groups categorized by the design of the study. RD = Risk Difference; CI = Confidence Interval; OR = Odd Ratio.

### Subgroup follow-up (Figure 9)

We perform a subgroup analysis according to follow-up duration, which is less or more than five years, with a random effect model (*I*^2^ = 54%). Six hundred fifteen patients with a follow-up of fewer than five years (279 cementless and 336 cemented TKA) were compared to 1037 patients with a follow-up longer than five years (414 cementless and 623 cemented TKA). Subgroup meta-analysis with a follow-up inferior to 5 years demonstrated no difference with RD_<5y_ = −0.011 CI95 (−0.03 to 0.003) (*p* = 0.64), while subgroup with more than five years of follow-up showed a significant difference in favor for cementless TKA, with RD_>5y_ = −0.025 CI95 (−0.04 to −0.009) (*p* = 0.045).

**Figure 9:**
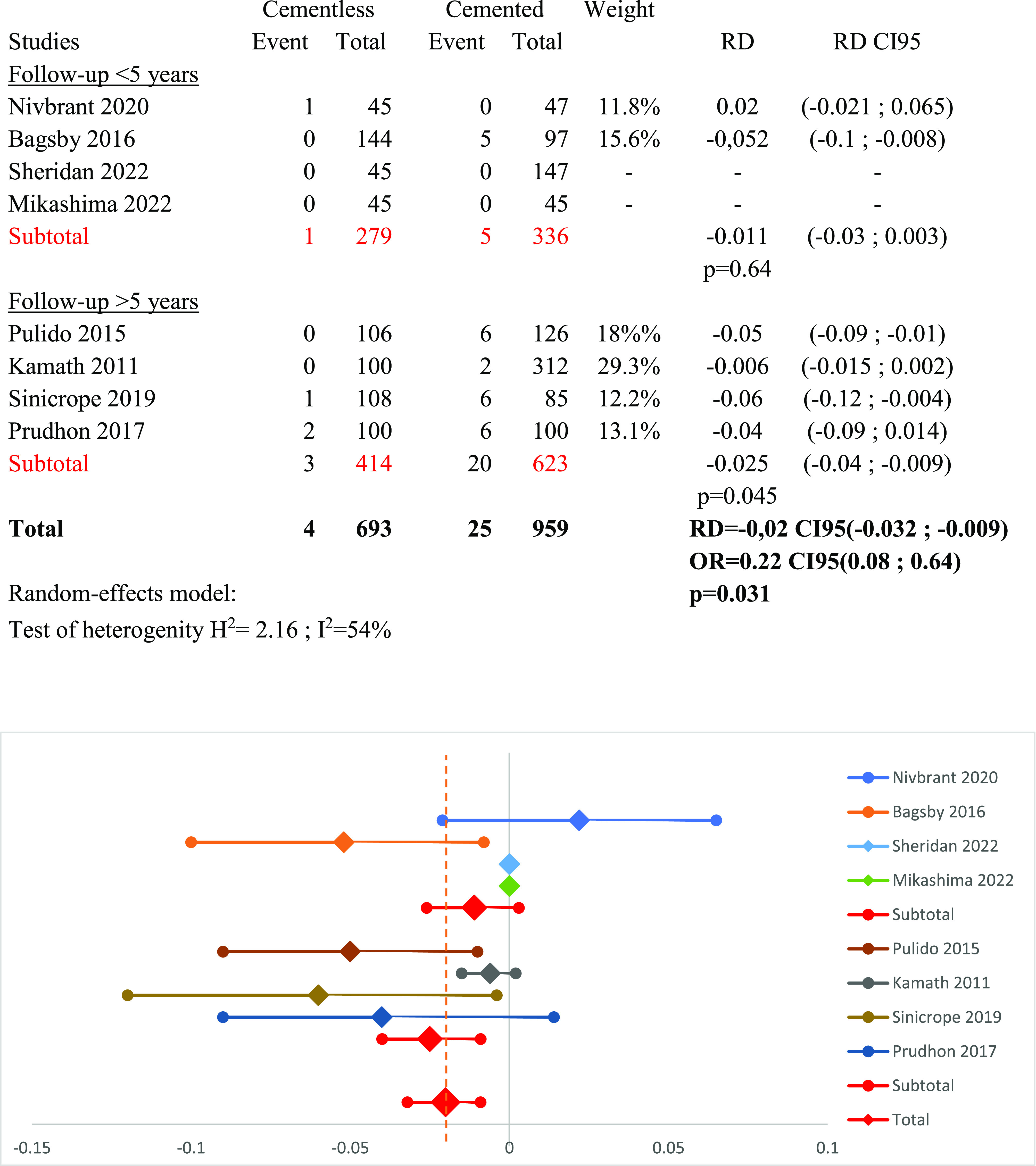
Forest plot demonstrating the risk difference of aseptic loosening between the cementless and cemented groups according to the duration of patient follow-up. RD = Risk Difference; CI = Confidence Interval; OR = Odd Ratio.

### Meta-analysis of radiolucent lines (Figure 10)

Four studies [1, 14, 15, 18] reported the incidence of radiolucent lines. Radiographic knee score was employed across all studies to assess these radiolucent lines. Using a random-effects model (*I*^2^ = 95%), no significant distinction emerged between the two groups: RD_RLL_ = 0.035 CI95 (−0.005 to 0.07) (*p* = 0.74).

**Figure 10:**
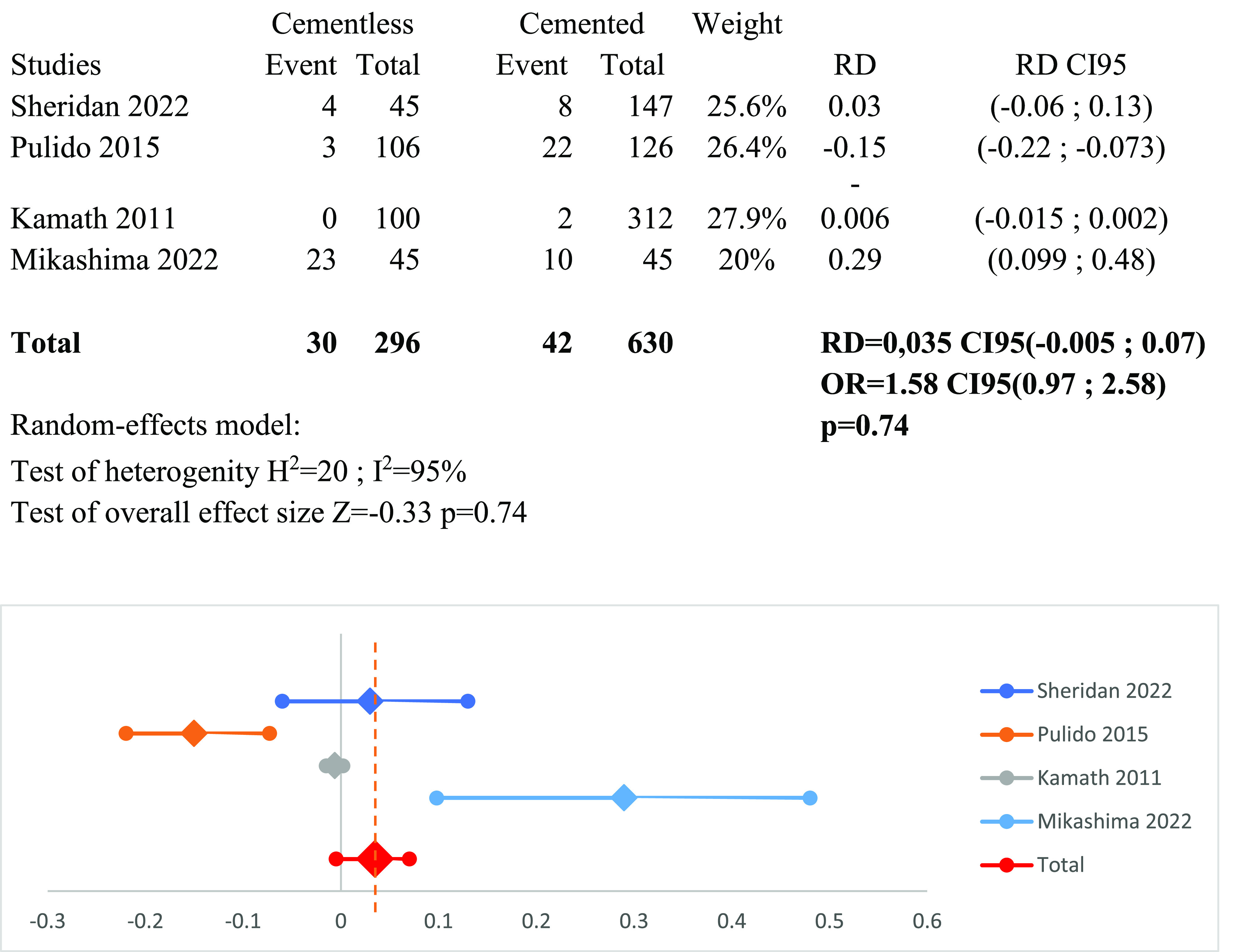
Forest plot demonstrating the risk difference of radio lucent lines between the cementless and cemented groups. RD = Risk Difference; CI = Confidence Interval; OR = Odd Ratio.

### Meta-analysis of infection and arthrofibrosis (Figures 11 and 12)

Four studies show an infection rate of 0.8% among cementless patients and 0.7% in cemented TKA patients. Following a fixed-effect analysis, no significant distinction was noted between the two groups regarding infection rate: RD_infection_ = 0.0002 CI95 (−0.01; 0.01) (*p* = 0.94).

**Figure 11:**
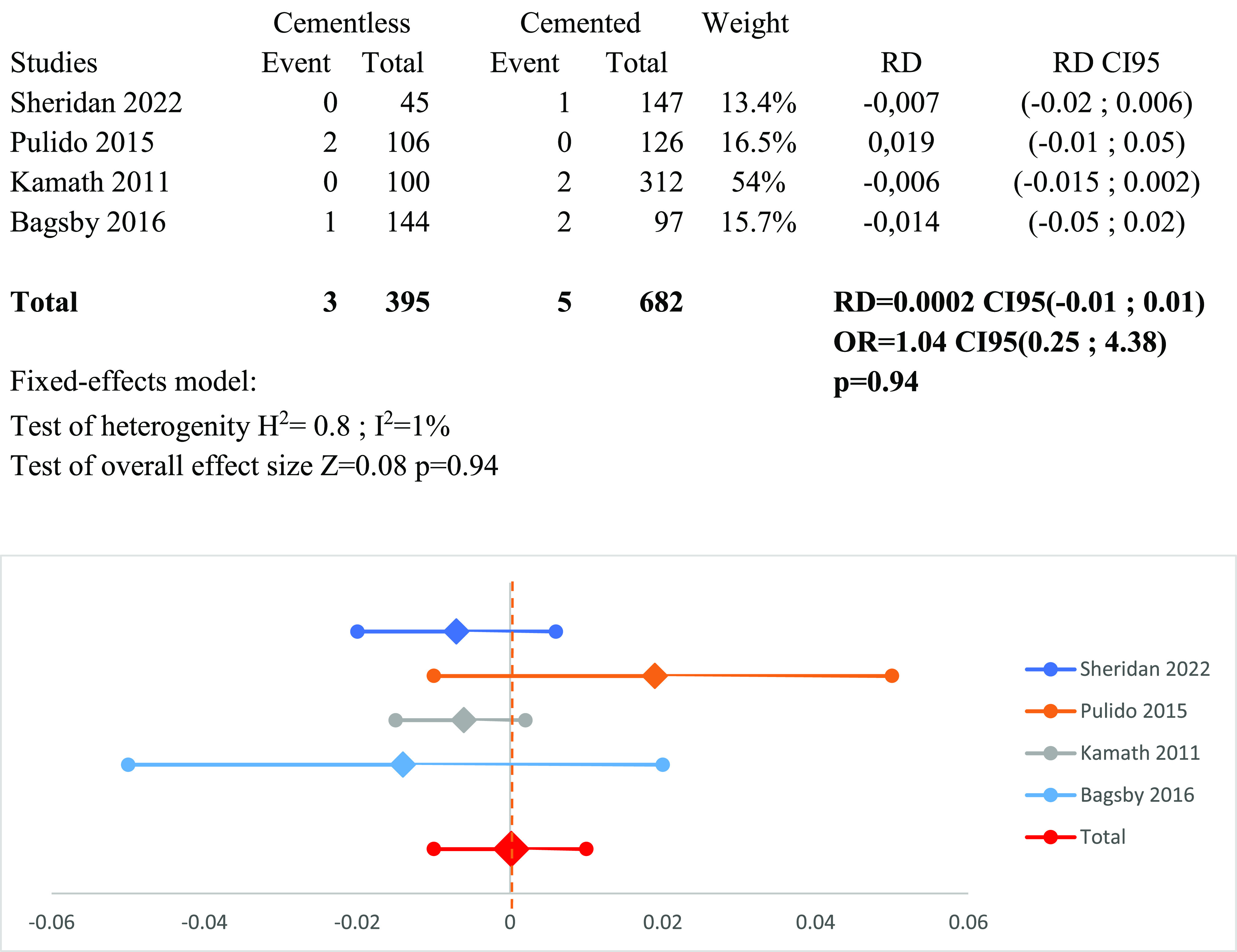
Forest plot demonstrating the risk difference of infection between the cementless and cemented groups. RD = Risk Difference; CI = Confidence Interval; OR = Odd Ratio.

**Figure 12:**
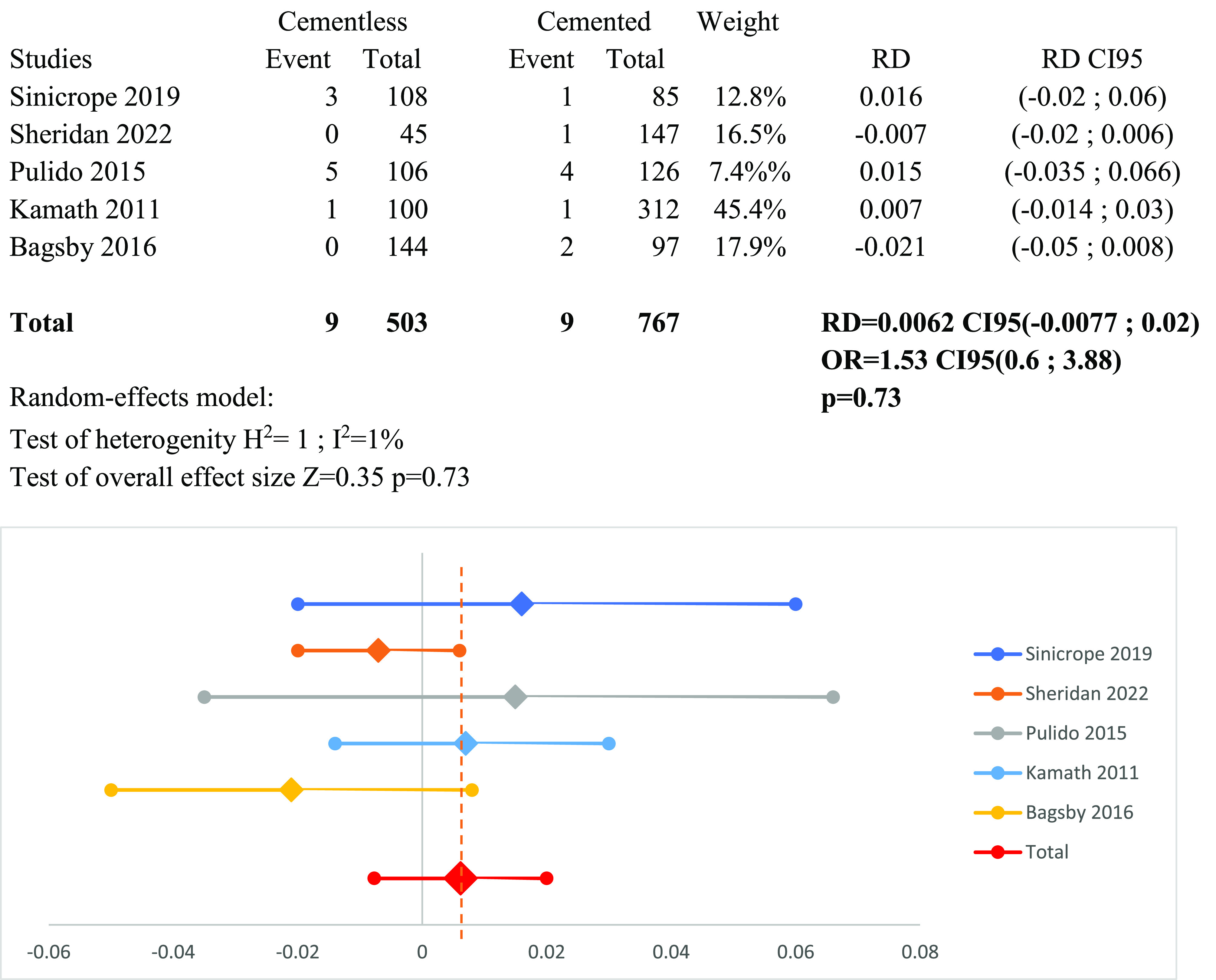
Forest plot demonstrating the risk difference of arthrofibrosis between the cementless and cemented groups. RD = Risk Difference; CI = Confidence Interval; OR = Odd Ratio.

Regarding arthrofibrosis, no notable difference was identified between the two groups. Arthrofibrosis occurred in 1.8% of patients in the cementless TKA group, while it was observed in 1.2% of the cemented Group: RD_arthrofibrosis_ = 0.0062 CI95 (−0.0077; 0.02) (*p* = 0.73).

## Discussion

Given the rising interest in non-cemented implants, our study is the first to present a specific comparative analysis of cemented and non-cemented posterior-stabilized implants. While numerous comparative studies or meta-analyses exist for cruciate retaining (CR) implants, none have focused on posterior-stabilized implants despite being the most commonly or second most frequently utilized implant in most total knee replacement registries [2, 3]. Our results suggest no difference between cemented and non-cemented posterior-stabilized prostheses. There might even be a superiority of non-cemented particularly in cases where surgeons opt for a stem or when dealing with obese patients.

Over the past ten years, the reasons for revising total knee prostheses have changed. While polyethylene wear was previously the primary cause of long-term revision in TKA, currently, aseptic loosening of implants is the leading cause of long-term failure in TKA. The hope of finding a more durable anchoring solution largely rests on non-cemented implants and new technologies (artificial intelligence and robotics) [20–23]. Recent research [10] has suggested that the survival rates of cementless TKA are on par with those of cemented TKA during short-term and medium-term follow-ups. Nevertheless, it remains imperative to conduct randomized long-term follow-up studies on both cemented and cementless posterior-stabilized TKA to establish conclusive insights regarding the preferred prosthesis fixation method for this TKA design.

The posterior stabilized configuration replaces the excised posterior cruciate ligament (PCL) with a cam mechanism. This design holds the potential for heightened stability when contrasted with PCL-retaining implants. Although widely favored, there are reservations surrounding the interaction between the cam and tibial plate, as it might transmit additional stresses through the polyethylene component. This stress can lead to the propagation of stresses across the junction between the implant and the bone, potentially escalating the susceptibility to wear and aseptic loosening. This situation prompts the query of whether the stability offered by cementless fixation is sufficiently robust to endure the supplementary stresses generated by the posterior stabilized layout. In 2019, Wojtowic et al. presented evidence showcasing the absence of translation, migration, or lack of stabilization over time in posterior-stabilized TKA [24]. The newly designed TKA component fixation surface exhibits a notably high coefficient of friction [25]. This surface also mimics cancellous bone in terms of porosity, pore size, and modulus of elasticity [26, 27].

Furthermore, it can encourage the attachment and mineralization of osteoblasts [28]. Notably, the similarity to cancellous bone also reduces the likelihood of triggering stress shielding [29]. These collective attributes foster successful fixation and may explain the comparable outcomes observed between cemented and cementless TKA. Mosich et al. demonstrated improved fixation in cementless TKA than in cemented at 16 months of follow-up [30].

The subgroup analysis highlights an absence of early survival differences between the two fixation methods but reveals an enhanced survival rate for the cementless group after a 5-year follow-up. This reinforces the hypothesis of an initially impacted cementless TKA survival rate due to early failures, which is subsequently maintained in the long term. This increase long-term survival could be attributed to the prosthetic bone integration capability and its increased resistance to fatigue failures compared to cemented TKA.

Publications on cementless TKA almost exclusively focus on unconstrained implants (CR). Some studies mentioned in this meta-analysis have compared cemented and non-cemented posterior-stabilized (PS) implants. However, no meta-analysis or registry analysis exists on this subject. However, this choice is not grounded in any scientific study demonstrating the superiority of unconstrained (CR) designs over constrained ones. Even though unconstrained implants offer satisfactory stability for the majority of operated knees, young patients with moderate joint deformities or a history of epiphyseal fractures often require PS TKA. It is precisely this population of young patients who need implants with long-term osseous anchorage. Our study does not allow us to determine whether non-cemented PS implants have a longer lifespan than unconstrained implants. However, it demonstrates a similar mid-term lifespan for non-cemented and cemented PS implants.

Concording with most prior investigations, our findings indicate that cementless fixation results in complication rates comparable to those of full-cemented fixation especially concerning radiolucent lines, arthrofibrosis, and complications. Regarding radiolucent lines, the Knee Society’s comprehensive scoring system for total knee arthroplasty radiographic assessment is extensively employed to gauge the presence and advancement of radiolucent lines. Our research revealed no discernible distinctions between cemented and cementless TKA methods. Nonetheless, our meta-analysis was limited to four studies investigating this particular outcome. In the context of cruciate-retaining (CR) design, various studies have brought attention to increased occurrences of radiolucent lines in cementless TKA compared to cemented implant approaches. Importantly, these findings do not appear to adversely affect functional outcomes or revision rates.

While certain studies had expressed concerns about an elevated risk of arthrofibrosis with cementless fixation, up to three times compared with cemented TKA, the available data to substantiate this remark was limited, and our analysis contradicts this notion.

Although this study adhered to stringent inclusion criteria, some limitations exist. Primarily, there is a scarcity of literature addressing PS cementless TKA. Moreover, the majority of available studies are retrospective and non-randomized. Given the limited number of studies, the overall study population is also modest, involving only 1652 patients, which contrasts with the global number of TKA procedures performed worldwide. This analysis could not include two studies with large populations [31, 32]. Indeed, these two studies compared non-cemented PS TKA with cemented PS TKA. However, these studies combined PS TKA and CR TKA. Despite several requests to the authors, we could not obtain detailed information regarding the outcomes of the PS TKA. These factors, combined with the inclusion of only English-language research, could potentially introduce bias to the meta-analysis. Our study does not provide a comparative analysis of the functional outcomes or compare different TKA designs between cemented and cementless TKA.

## Conclusion

Our study is the first meta-analysis on the survival and complication rates when comparing cementless and cemented versions of posterior stabilized implants. It does not enable us to assert the long-term superiority of non-cemented PS implants. We observed comparable rates for cemented and cementless posterior-stabilized TKAs over a medium-term follow-up period. These reassuring mid-term results regarding the use of non-cemented posterior stabilized (PS) implants should be validated through registry analyses and long-term prospective randomized studies for further confirmation and to ascertain the safety of cementless posterior-stabilized TKA.

## Data Availability

There is no data available.
